# An Optimized High-Throughput Immuno-Plaque Assay for SARS-CoV-2

**DOI:** 10.3389/fmicb.2021.625136

**Published:** 2021-02-12

**Authors:** Alberto A. Amarilla, Naphak Modhiran, Yin Xiang Setoh, Nias Y. G. Peng, Julian D. J. Sng, Benjamin Liang, Christopher L. D. McMillan, Morgan E. Freney, Stacey T. M. Cheung, Keith J. Chappell, Alexander A. Khromykh, Paul R. Young, Daniel Watterson

**Affiliations:** ^1^School of Chemistry and Molecular Biosciences, The University of Queensland, St Lucia, QLD, Australia; ^2^The Australian Institute for Biotechnology and Nanotechnology, The University of Queensland, St Lucia, QLD, Australia; ^3^Australian Infectious Disease Research Centre, The University of Queensland, St Lucia, QLD, Australia

**Keywords:** coronaviruses, SARS-CoV-2, immuno-plaque assay (iPA), viral quantification

## Abstract

Severe acute respiratory syndrome coronavirus-2 (SARS-CoV-2) has been identified as the causative agent of coronavirus disease 2019 and is capable of human-to-human transmission and rapid global spread. The rapid emergence and global spread of SARS-CoV-2 has encouraged the establishment of a rapid, sensitive, and reliable viral detection and quantification methodology. Here, we present an alternative assay, termed immuno-plaque assay (iPA), which utilizes a combination of plaque assay and immunofluorescence techniques. We have extensively optimized the conditions for SARS-CoV-2 infection and demonstrated the great flexibility of iPA detection using several antibodies and dual-probing with two distinct epitope-specific antibodies. In addition, we showed that iPA could be utilized for ultra-high-throughput viral titration and neutralization assay within 24 h and is amenable to a 384-well format. These advantages will significantly accelerate SARS-CoV-2 research outcomes during this pandemic period.

## Introduction

The world is currently experiencing one of the most significant viral pandemics of the modern era. Health authorities were first alerted on December 31, 2019 to the emergence of a novel respiratory illness in the city of Wuhan, China (ProMED, 2019). Sequencing results obtained from patients presenting with an unknown viral pneumonia identified a novel coronavirus (nCoV-19) phylogenetically related to the severe acute respiratory syndrome coronavirus (SARS-CoV) (Zhou et al., 2019; Wu et al., 2020). The nCoV-19 was subsequently renamed as SARS-CoV-2, which is the causative agent of coronavirus disease 2019 (COVID-19) and is capable of human-to-human transmission and rapid global spread (World Health Organization WHO, 2020b, c). By September 28, 2020, SARS-CoV-2 has infected 32.7 million across the globe, with more than 991,000 deaths at an estimated fatality rate of 3% (World Health Organization WHO, 2020a). According to the Chinese Center for Disease Control and Prevention, 81% of the COVID-19 disease were characterized by mild flu-like symptoms (Wu and McGoogan, 2020). However, 14% of hospitalized patients have more severe disease outcomes such as dyspnea (shortness of breath), respiratory frequency lower than 30/min, and blood oxygen saturation lower than 93%. Additionally, 5% of patients were admitted to intensive care units, presenting with respiratory failure and multiple organ dysfunction or failure (Wu and McGoogan, 2020).

Severe acute respiratory syndrome coronavirus-2 belongs to the genus *Betacoronavirus*, a large, enveloped, non-segmented positive-sense RNA virus with a complex genome of approximately 30 kb. Similarly to SARS-CoV, the genome of the novel coronavirus is organized in six major open-reading frames (ORFs), encoding for 16 non-structural proteins (NS) and four structural proteins named as spike (S), envelope (E), membrane (M), and nucleocapsid (N). In addition, there are a number of other ORFs present between the structural proteins which encode for accessory genes (Zhou et al., 2019; Wu et al., 2020). The S protein is the major surface glycoprotein of the virus particle (Lan et al., 2020; Wrapp et al., 2020). During viral infection, the S protein is cleaved into S1 and S2 subunits. The S1 subunit contains the receptor-binding domain (RBD), which recognizes angiotensin-converting enzyme 2 (ACE2) as its early receptor. The S2 subunit contains another cleavage site and is cleaved by host protease that is critical for viral infection (Li et al., 2003; Simmons et al., 2004; Madu et al., 2009; Walls et al., 2020).

The rapid emergence and global spread of SARS-CoV-2 has spurred tremendous scientific efforts to develop diagnostic, antiviral, and vaccine countermeasures. As such, the establishment of a rapid, sensitive, and reliable viral detection and quantification methodology is critical. Several methods have thus far been employed for infectious virions, including the classical plaque assay as well as tissue culture infectious dose (TCID_50_) (Svensson et al., 1999; Brien et al., 2013; Smither et al., 2013; Agbulos et al., 2016; Karakus et al., 2018; Smith et al., 2019; Amanat et al., 2020; Mendoza et al., 2020). Here, we present an alternative assay, termed immuno-plaque assay (iPA), which utilizes a combination of plaque assay and immunofluorescence techniques, and show that it allows the determination of viral titer/inhibition within 24 h and is amenable to high-throughput format. These advantages have the capacity to accelerate SARS-CoV-2 research in this pandemic.

## Materials and Equipment

### Essential Equipment and Software

- Odyssey Infrared Imaging System infra-red high-resolution scanner LI-COR CLX
- Image Studio Software
- R Studio
- Viridot

### Preparation of Reagents

(1) 2% carboxymethylcellulose sodium stock (CMC), medium viscosity (Sigma-Aldrich, Cat. No. C4888-1KG)
  - Fill a 500-ml glass bottle with 300 ml of Milli-Q water.
  - Weigh and add 6 g of CMC powder into the glass bottle, being careful not to deposit too much powder on the side of the bottle.
  - Allow the CMC powder to dissolve overnight in a shaking incubator at 220 rpm, 37°C.
  - On the next day, autoclave to sterilize the CMC stock and store at 4°C.
(2) 2X Medium 199, Earle’s Salts, powder (Gibco, Thermo Fisher Scientific, Cat. No. 3100-035)
  - In a sterile 500-ml glass bottle, add 500 ml of autoclaved Milli-Q water.
  - Add 9.5 g of M199 (powder) and 2.2 g of NaHCO_3_ and mix with a sterile magnetic stirrer bar.
  - Once dissolved, filter the media using a Corning 500-ml Vacuum Filter/Storage Bottle System (Corning, Cat. No. CLS431097) and store at 4°C.

Note: Before using the 2X M199 media (500-ml stocks), add 25 ml of heat-inactivated (HI) fetal calf serum (FCS; Bovogen, United States) and 5 ml of penicillin-streptomycin (P/S) (10,000 U/ml) and store at 4°C.

(3) Overlay medium
  - Before preparing the overlay medium, the 2% CMC and 2X M199 (containing 5% FCS and P/S) need to be pre-warmed at 37°C for 30 min.
  - In a sterile 500-ml glass bottle, add one part of 2% CMC to one part of 2X M199.
  - Mix by inversion (avoid bubbles) until obtaining a homogeneous solution and keep at 37°C until use.

Note: It is preferential to prepare fresh overlay medium.

(4) Blocking buffer
  - KPL Milk Diluent/Blocking Solution Concentrate (Sera care, United States, Cat. No. 5140-0011).
  - Pierce Clear Milk Blocking buffer (Thermo Fisher Scientific Inc., United States, Cat. No. 37587).
  - Phosphate-buffered saline (PBS; pH 7.4).
  - Tween-20.
(5) Preparation of primary antibody
  - Prepare 1 mg/ml of the primary antibody stock in 50% glycerol solution and mix by vortexing for 20 s. Spin down and store at −20°C. For probing the iPA plates, dilute the primary antibody 1/1,000 in fresh blocking solution and use 50 or 20 μl/well (50 or 20 μg/well) for 96- and 384-well plates, respectively.
(6) Reconstitution of IRDye^®^ secondary antibody (0.5 mg)
  - Prepare 50% glycerol solution in Milli-Q water.
  - Add 0.5 ml of 50% glycerol solution; mix by inverting the tube.
  - Incubate at 22°C in the dark for 30 min to allow rehydration.
  - Store at −20°C.
  - For probing the iPA plates, dilute the antibody 1/2,500 (400 ng/ml) in fresh blocking solution and use 50 or 20 μl/well (20 or 8 ng/well) for 96- and 384-well plates, respectively.
(7) Washing buffer

Phosphate-buffered saline (pH 7.4) containing 0.05% Tween-20.

## Methods

### Cell Lines

Vero76 and VeroE6 cells (African green monkey kidney cell clones) were cultured in Dulbecco’s modified Eagle’s medium (DMEM) supplemented with 10% HI fetal calf serum (FCS) (Bovogen, United States), penicillin (100 U/ml), and streptomycin (100 μg/ml) and maintained at 37°C with 5% CO_2_.

### Viral Isolate

An isolate of SARS-CoV-2 named QLD02 (GISAID accession EPI_ISL_407896) was recovered from a patient’s nasopharyngeal aspirate following inoculation of VeroE6 cells. The passage 2 QLD02 virus was provided by Queensland Health Forensic and Scientific Services, Queensland Department of Health. Viral stock (passage 3) was then generated from the obtained passage 2 virus on VeroE6 cells and stored at −80°C. Virus stock titer was determined by iPA.

### Generation of Monoclonal Antibodies

Recombinant monoclonal antibodies (mAb) that recognize SARS-CoV-2, including S309, CB6, CR3022, and 9A1, were generated in Chinese hamster ovary (CHO) cells (ter Meulen et al., 2006; Chi et al., 2020; Pinto et al., 2020; Shi et al., 2020). Briefly, the variable regions for each of the antibodies were codon-optimized and synthesized for *Cricetulus griseus* (hamster) cell expression by Integrated DNA Technologies (Singapore) and cloned in-frame into plasmids encoding the human or mouse constant heavy- and light-chain IgG1 backbone as described previously (Jones et al., 2010). The CHO cells were co-transfected with plasmids encoding the heavy and light chain for each antibody, and at 7 days post-transfection, the antibody was purified with a protein A column (GE healthcare). The antibodies were validated by SDS-PAGE and ELISA before use (data not shown).

### Tissue Culture Infectious Dose (TCID_50_)

A day before infection, approximately 4 × 10^4^ cells per well were seeded in a 96-well plate and incubated overnight to reach 100% confluency. The virus was serially diluted 10-fold in DMEM (supplemented with 2% of FCS and P/S), and immediately 100 ul of each dilution was added onto the cells. At 3 days post-infection, the inoculum was removed, and the cells were fixed with 4% formaldehyde in PBS for 2 h at room temperature and stained for 1 h with 0.2% crystal violet solution (80% of PBS and 20% methanol) to reveal the cytopathic effects. Then, the crystal violet solution was removed, and the plates were washed five times with tap water and fully dried at room temperature. Fifty percent endpoints were calculated using Reed and Muench’s (1938) calculation and expressed as tissue culture infectious dose (TCID_50_)/ml. Three independent experiments with six replicates were performed to determine the viral titers by TCID_50_.

### Plaque Assay

The standard plaque assay (PA) was performed on VeroE6 cells. Briefly, 1 × 10^6^ cells per well were grown in six-well plates and infected with 10-fold serial dilutions of the viruses for 30 min at 37°C, and subsequently, 2 ml of an overlay medium was added. The overlay medium contains a final concentration of 0.375% low-melting point agarose in 5% HI FCS-DMEM medium. At 3 days post-infection, the cells were fixed with 4% formaldehyde in PBS for 2 h at room temperature before the overlay medium was removed, and the cells were stained with 0.2% crystal violet solution (80% of PBS and 20% methanol). The cells were then washed to reveal the plaques. The result was expressed as plaque-forming units (PFU)/ml, with a limit of detection of 50 PFU/ml.

### Growth Kinetics

Severe acute respiratory syndrome coronavirus-2 replication kinetics was assessed on VeroE6 and Vero76 cells. Briefly, approximately 1 × 10^6^ cells per well were seeded in six-well plates 1 day before infection. The cells were infected at a multiplicity of infection (MOI) of 0.1 for 30 min at 37°C with rocking every 10 min. The monolayer was washed thrice with 1 ml of additive-free DMEM, and finally 3 ml of DMEM (supplemented with 2% FCS and P/S) was added; the cells were maintained at 37°C with 5% CO_2_. Infectious viral titers were quantified from supernatant harvested at the indicated time points: 0, 1, and 2 days post-infection. The viral titer was determined by iPA on VeroE6 cells. Three independent experiments were performed.

### Statistical Analysis

All data were analyzed using Prism software (GraphPad 8.1.2). For growth kinetics, to compare within groups, a multiple comparison using two-way ANOVA test with Sidak’s correction was used. The level of statistical significance was set at 95% (*p* = 0.05). For plaque reduction neutralization test (PRNT), to determine the EC50 value, best-fit curve was tested using non-linear regression with inhibitor *vs*. response (three parameters) model.

**FIGURE 1:**
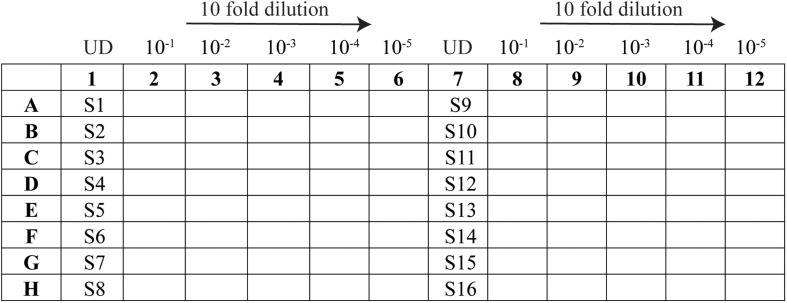
Schematic representation of the 96-well layout of sample preparation for viral titration. Samples 1–16 are represented as S1–S16 and the undiluted samples as UD.
  - In the sample lanes (columns 1 and 7), add 55 μl of each virus sample to be tested (minimum volume required is 45 μl).
  - In the remaining wells, add 180 μl of DMEM supplemented with 2% FCS and P/S.
  - Using an eight-channel pipette, take 20 μl of supernatant from the sample well (column 1) and pipette into “column 2”; mix by pipetting at least 20 times. Repeat for column 2 to column 3, changing tips in between, until column 6.
  - Perform the same for the replicate on the right side of the plate.
  - Once the 10-fold serial dilution is completed, proceed to the next step or keep at 4°C.
(C.) Infection
  - Adjust an eight-channel pipette to a volume of 25 μl for a 96-well plate or 10 μl for a 384-well plate.
  - Take iPA plates seeded with Vero cells out from the incubator.
  - Remove media by flicking out into a container lined with absorbent paper to reduce splashing.
  - Transfer from the 10-fold serial dilution plate into the iPA plate containing the cells, following the same layout. Use 25 or 10 μl of the sample for a 96- or 384-well plate, respectively.
  - Moving from right to left (starting from column 6), take the inoculum and add into the same column of the iPA plate; continue through columns 5, 4, 3, 2, and 1 without changing tips.
  - Change new tips when starting on column 12 and continue through to column 7.
  - Place the iPA plate into an incubator at 37°C and allow the cells to be infected for 15, 30, or 60 min. No rocking is required.
  - While waiting, prepare the CMC overlay medium.
  - Mix one part of 2% carboxymethyl cellulose to one part of 2 × M199 media (prepare 30 ml of overlay per iPA plate).
  - After the required time for infection, use an eight-channel pipette to add 175 or 50 μl of overlay per well to a 96- or 384-well plate, respectively. No need to remove the virus inoculum.
  - Place the iPA plates back into the incubator at 37°C with 5% CO_2_ and incubate for 14 h (overnight).
(D.) Fixing the plates
  - At 14 h post-infection, remove the plates from the incubator.
  - Using a 12-channel pipette set to 200 μl, remove the overlay medium from the plates. No need to change tips.
  - Submerge the entire plate in ice-cold 80% acetone.
  - Allow the cells to fix in −20°C freezer for 30 min.
  - Flick the acetone out of the plates into a container in the fume hood.
  - Dry the plates fully for 2 h (quicker on the hood grate). It is essential to highlight that the fixation process is not finished until the cell monolayer is completely dry.
  - Store the dried plates at room temperature or proceed to the next section.
(E.) Probing the plates with antibodies

In this manuscript, we are providing a step-by-step procedure of an optimized iPA protocol for viral titration and PRNT:

Protocol 1. iPA, an overview of this protocol is provided in Supplementary Material 1.

(A.) Seeding the plates for iPA (1 day before)
  - Prepare 11 ml of Vero cells (cell density of 4 × 10^5^ cells/ml in DMEM supplemented with 10% FCS and P/S) for each 96- or 384-well plate as required.
  - Using a multichannel pipette, fill the plates with a volume of 100 or 50 μl per well for a 96- or 384-well plate, respectively.
  - Place the plates at 37°C incubator with 5% CO_2_ and incubate overnight.
  - Sample preparation
(B.) Prepare the dilution plate (96-well plate) as per the layout in Figure 1:

We have observed that the background can be further reduced by blocking the plates with Pierce Clear Milk blocking buffer (Thermo Fisher Scientific Inc., United States, Cat. No. 37587) or KPL (Cat. No. 5140-0011). Dilute the concentrated blocking solution in 1 × PBS containing 0.05% Tween 20 (PBS-T). Dilute the primary and secondary antibodies in blocking solution.

- Add 150 or 50 μl per well of blocking solution (for a 96- or 384-well plate, respectively) and incubate at room temperature or 37°C for 60 min. Alternatively, the blocking step could be performed with overnight incubation at 4°C.
- Remove the blocking buffer and tap the plates to remove excess.
- Dilute the primary antibody (1/1,000) in blocking solution, add 50 or 20 μl/well for a 96- or 384-well plate, respectively, and incubate for 60 min at 37°C.
- After primary incubation, wash the monolayer three to five times with PBS-T and incubate for 5 min for each wash at room temperature. After the last wash, tap the plates to remove the excess of the washing buffer.
- Dilute the IR dye conjugated secondary antibodies (1/2,500) in blocking solution, add 50 or 20 μl/well for a 96- or 384-well plate, respectively, and incubate for 60 min at 37°C.
- After secondary incubation, wash the monolayer as performed for the primary incubation.
- Dry the plates (avoid light exposure).

(F.) Scanning the plates on the infra-red imager
  - We use the Image Studio software that is provided with the LICOR Odyssey Scanner.
  - Wipe the iPA plates and glass clean using Kimwipes.
  - On the Image Studio Lite software, choose the settings:
    For a 96-well plate:
    a. Turn on Channel 800 or 700 or both
    b. Channel intensities – set to auto
    c. Resolution – set to 42 μm
    d. Quality –medium
    e Focus – 3 and 2.75 mm for a 96- and 384-well plate, respectively
  - Select the scan area and press “Start.”
(G.) Counting and tabulating the virus titers

Software:

- “Mousotron” – mouse clicker counter
- “Snip and Sketch” – screen capture software (Windows)
- Using the Snip and Sketch app, take a screenshot of the iPA plate.
- Open Mousotron to record the number of mouse clicks.
- Switch back to Snip and Sketch and click on every focus (hence, marking each spot with a red dot).
- Record the number of foci counted and record which dilution that was counted in.

**FIGURE 2:**
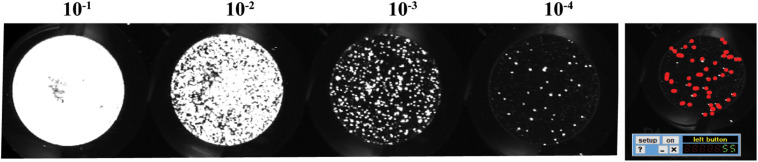
Example of scanned plate performed by immuno-plaque assay showing the 10-fold serial dilution in the 96-well format and foci numbers counted by Mousotron.

Tip: To get the most accurate titers, count wells that have between 20 and 80 foci (Figure 2).

Alternatively, an automated virus plaque (immunofocus) counter, named as Viridot (Katzelnick et al., 2018), could perform this analysis and will be further explained in the protocol 2 section.

(H.) Viral titer calculation (FFU/ml)

Taking the above example.

55 foci counted in dilution 10^–4^ (dilution factor is 0.0001), and the inoculum volume was 0.025 ml.

(1) $$\mathrm{Virus titer}=\frac{\text{No}.\mathrm{of}\mathrm{foci}\mathrm{counted}\mathrm{in}\mathrm{the}\mathrm{well}}{\mathrm{Dilution}\mathrm{counted}\mathrm{foci}\times \mathrm{Amount}\mathrm{of}\mathrm{inoculation}\mathrm{volumn}\left(\mathrm{in}\mathrm{ml}\right)}$$

Example:

$$\frac{55}{0.0001\times 0.025}=22,000,000\text{FFU}/\text{mL}$$

**FIGURE 3:**
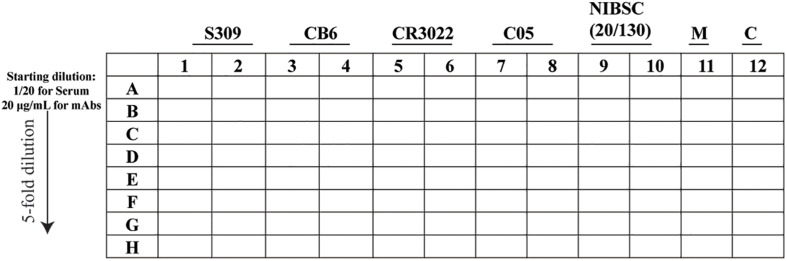
Schematic representation of the 96-well layout of sample preparation for the plaque reduction neutralization test. The uninfected cells are represented as M and the virus control as C.
  - In lane A, prepare the starting dilution for the serum and the monoclonal antibody (mAb) in DMEM containing 5% FCS and P/S, adjusted to a final volume of 100 or 20 μl for a 96- and 384-well plate, respectively, and then mix by pipetting at least 10 times.
  - In the remaining wells, add DMEM supplemented with 5% FCS and P/S (80 or 16 μl for a 96- and 384-well plate, respectively).
  - Using a 12-channel pipette, take the sample (20 or 4 μl for a 96- and 384-well plate, respectively) from line A and pipette into “line B” and mix by pipetting at least 10 times.
  - Repeat for line B to line C, remembering to change tips in between, until line H.
  - Once the fivefold serial dilution is completed, using a 12-channel pipette, transfer 30 or 10 μl, for a 96- and 384-well plate, respectively, of the fivefold serial dilution into a new plate and proceed to the next step or keep at 4°C.
(B.) Virus and antibody/sera complex
  - Virus solution preparation:

Protocol 2. PRNT – an overview of this protocol is provided in Supplementary Material 1.

(A.) Sample preparation
  - Heat the inactivated serum at 56°C for 30 min (This step is only for the serum sample).
  - Prepare the dilution (96-well plate) as per the layout in Figure 3.

For a 96-well plate format: in order to obtain approximately 60 immuno-plaques/well, prepare a solution containing 6 × 10^3^ FFU/ml^∗^ and use 30 μl to mix with the antibody dilutions.

For a 384-well plate, prepare a solution containing 1.2 × 10^4^ FFU/ml^∗^ and use 10 μl to mix with the antibody dilutions. Prepare all the virus solutions in DMEM supplemented with 2% FCS and P/S.

- Virus and antibody/sera complex: using a 12-channel pipette, add virus (30 or 10 μl of virus solution for 96- and 384-well plates, respectively) to each well of plates containing fivefold serial dilutions of antibodies and mix by pipetting at least five times. It is important to note that the 96- and 384-well plates will contain a final volume of 60 or 20 μl per well for the 96- and 384-well plates, respectively.
- Incubate the complex (virus and antibody/mAbs) at 37°C for 60 min.
- Add the virus and antibody/mAbs complex onto pre-seeded Vero E6 (50 or 15 μl for 96- and 384-well plates, respectively) and incubate at 37°C for 30 min.
- After the infection step, use a multichannel pipette to add 175 μl of overlay per well or 50 μl for a 384-well plate. For this PRNT, removal of the virus–antibody complex is not necessary.
- Place the iPA plates back into the incubator at 37°C with 5% CO_2_ and incubate for 14 h.
- Plates were fixed and probed, similarly as the iPA protocol (see the above section of protocol 1, steps D and E).

^∗^Note: viral titration was determined on a 96-well format.

(C.) Counting the immuno-plaques using an automated virus plaque counter Viridot

(C.1) Creating individual images of each well:

Note: Before starting, make sure to have downloaded and installed the Viridot software as per the instructions in the published paper (Katzelnick et al., 2018).

**FIGURE 4:**
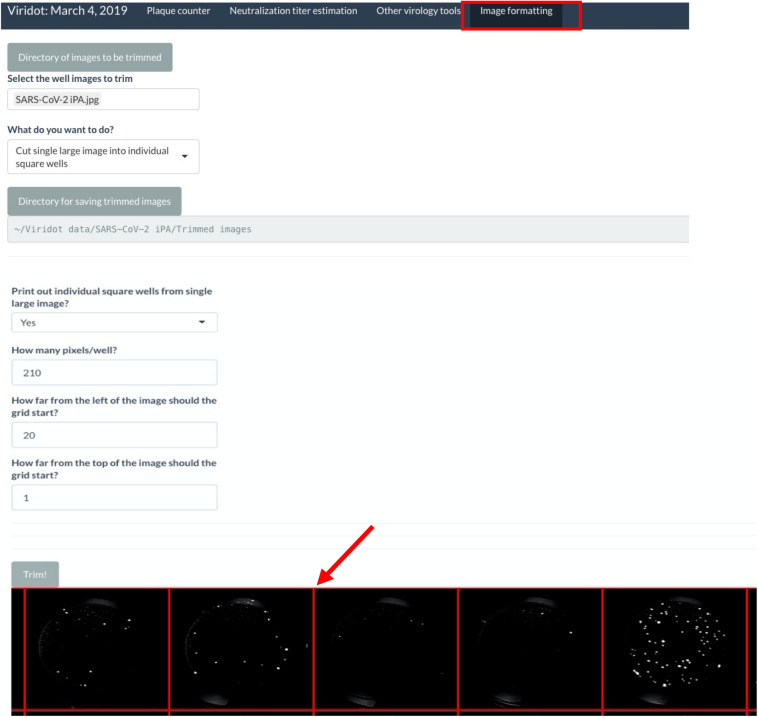
Screenshot of Viridot program highlighting the different options within the “Image Formatting” tab.
- Click on “Directory of images to be trimmed” and navigate to the folder where the plate image is saved.
- Within that folder, select the image to be trimmed.
- Under “What do you want to do?”, select “Cut single large image into individual square wells.”
- Click on “Directory for saving trimmed images” and navigate to where you want to save your trimmed images. Note that this should be a folder containing only the trimmed images, so create a new folder named “Trimmed images.”
- Under “Print out individual square wells from a single large image?”, keep the selection as “no.”
- Next, click “Trim!”, and a preview of the plate image with an overlay of the square wells will appear. Alter the “How many pixels/well?”, “How far from the left of the image should the grid start?”, and “How far from the top of the image should the grid start?” parameters until the individual squares in the overlay line up with each well of your 96-well plate image.
- Once the overlay is appropriately aligned, change the “Print out individual square wells from single large image?” option to “yes” and click “Trim!” The individual wells will now be saved in the previously defined directory.
- (C.2) Counting the plaques from individual image wells:

- Export the image as TIFF or JPEG, with a resolution of 600 dots per inch, from the Image Studio software.
- If scanning multiple plates at once, use the Snip and Sketch software to take a screenshot of each plate individually, and save the images.
- Launch Viridot as per the published instructions and navigate to the “Image formatting” tab (Figure 4).

**FIGURE 5:**
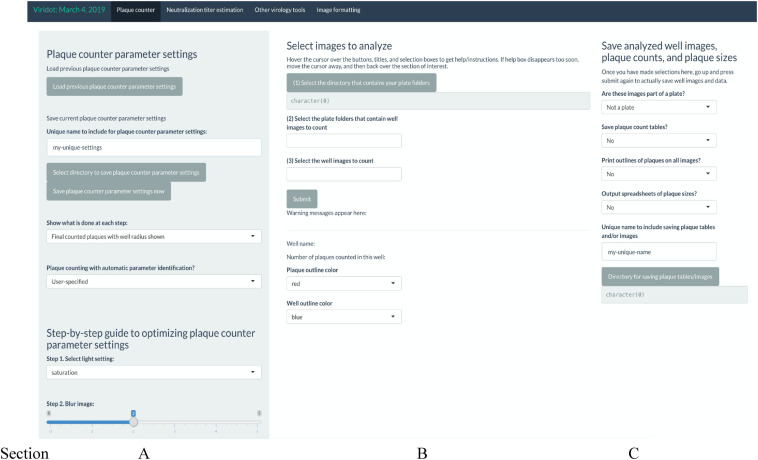
Screenshot of Viridot program showing the three main sections within the “Plaque Counter” tab.

In Viridot, navigate to the “Plaque counter” tab along the top of the program. There are three main sections that require adjustment of the settings before use (Figure 5):

(A) Under “Select images to analyse”, go through options (1), (2), and (3) in order to select the directory that contains your plate folder, select the plate folders that contain the well images (i.e., the “Trimmed images” folder), and then select an example image to count from that folder. Note that it is best to choose an image that contains 20–80 foci to ensure that the parameter settings are optimized for each plate image.

(B) Under “Plaque counter parameter settings”, change “Plaque counting with automatic parameter identification?” drop-down menu to “User-specified.” Then, change the settings for each parameter to the following values (note: these settings may need to be optimized for the individual plate scans):

• Step 1: select light setting = green
• Step 2: blur image: = 0
• Extra option: Remove strings/fibers in image = 0
• Step 3.1: cut well edges = 0
• Step 3.2: insert value for pixels outside the well = black (0)
• Step 4.1: apply contrast to image based on background intensity = 1
• Step 4.2: apply contrast to image based on plaque intensity = 1
• Step 5.1: select difference in pixel value to distinguish plaque from background = 0.12
• Step 5.2: select the size (in pixels) of the window for applying the thresholding algorithm to the image = 10
• Step 6: dilate your plaques to ensure they are counted as single plaques = 3
• Step 7: cut overlapping plaques so that they are counted separately = 1
• Step 8.1: define the minimum pixel size to count as a plaque = 0
• Step 8.2: define the maximum pixel size to count as a plaque = 10,000

**FIGURE 6:**
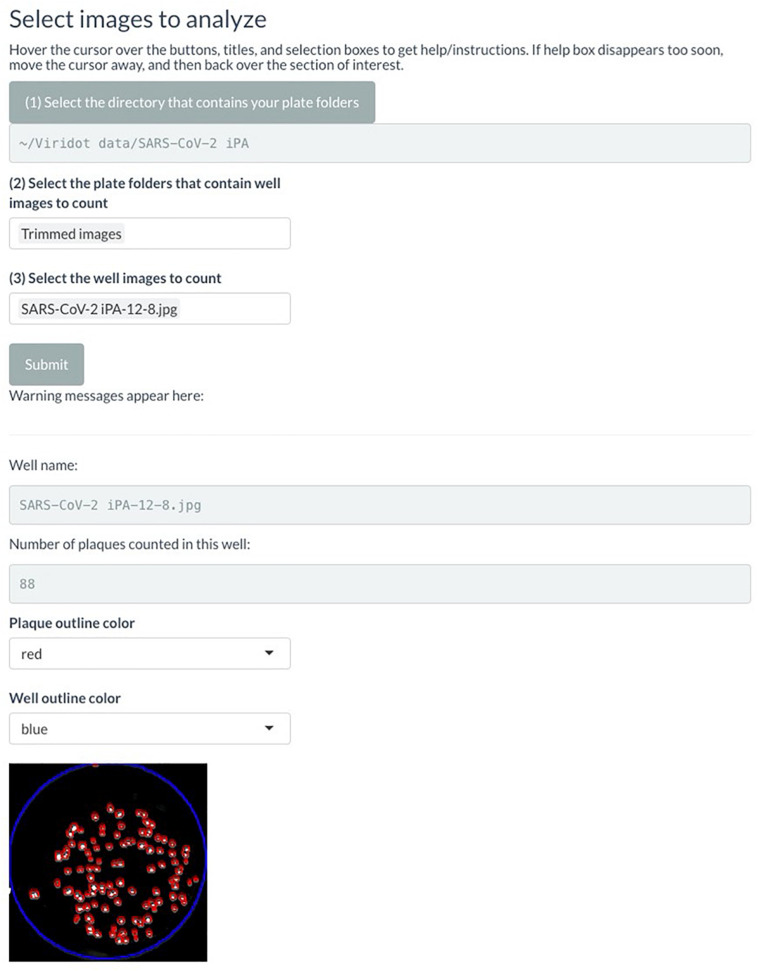
Screenshot of Viridot program showing section B of “Plaque Counter” tab after adjusting the parameter for analysis.

Click “Submit” and check that the red outlines of the counted foci are appropriate (Figure 6).

If not satisfied with the program’s counting of the foci, alter the parameters until the counting is appropriate. Utilize the “Show what is done at each step” drop-down menu to see the progress of the image parameters at each step.

**FIGURE 7:**
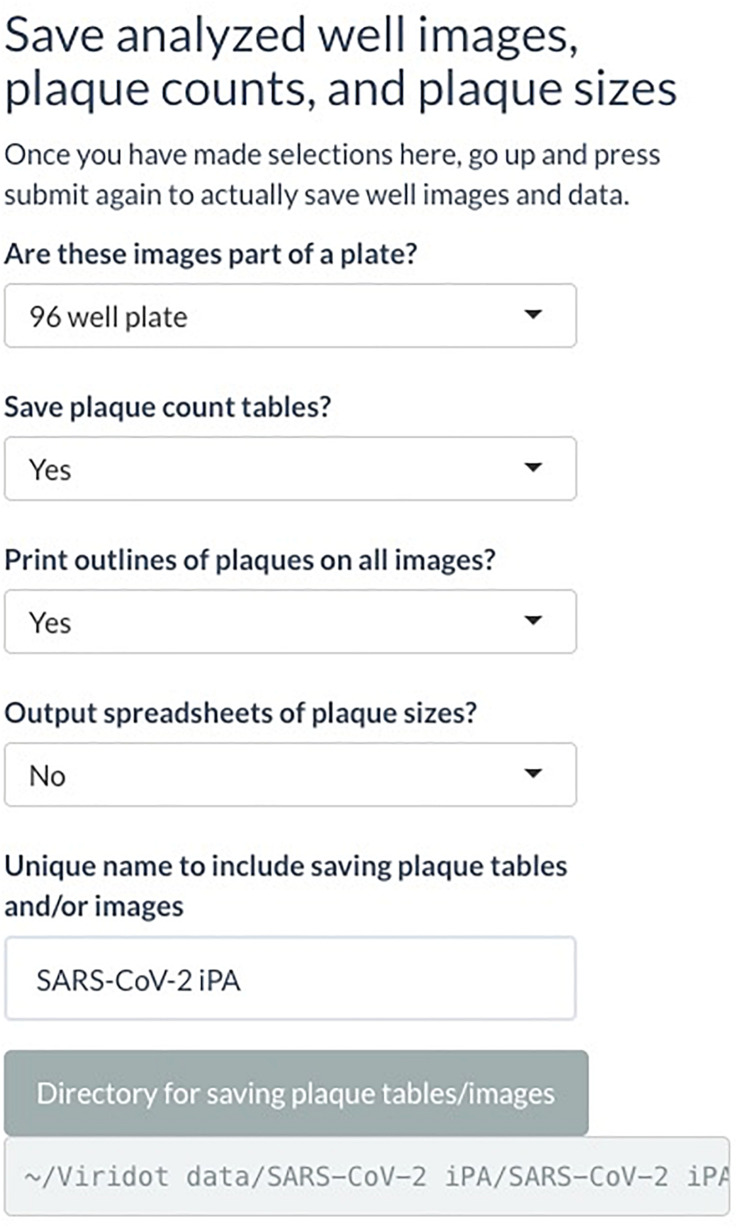
Screenshot of Viridot program showing section C of “Plaque Counter” tab after adjusting the parameter for analysis.

Once satisfied with the program’s foci counting, under option (3) in section A, delete the test well that was selected and select “all” instead.

(C) Under “Save analyzed well images, plaque counts, and plaque sizes” drop-down menus, if you want to save the plaque count table, the individual well images with the foci outlined, and the foci sizes. A unique name can also be added here. Select the directory where the data will be saved (Figure 7).

Click “Submit.”

Navigate to the directory where the plaque tables/images were saved and open the plaque table Excel spreadsheet to see the foci numbers in each well of the 96- or 384-well plates.

## Results

### Optimization of an iPA for SARS-CoV-2

**FIGURE 8:**
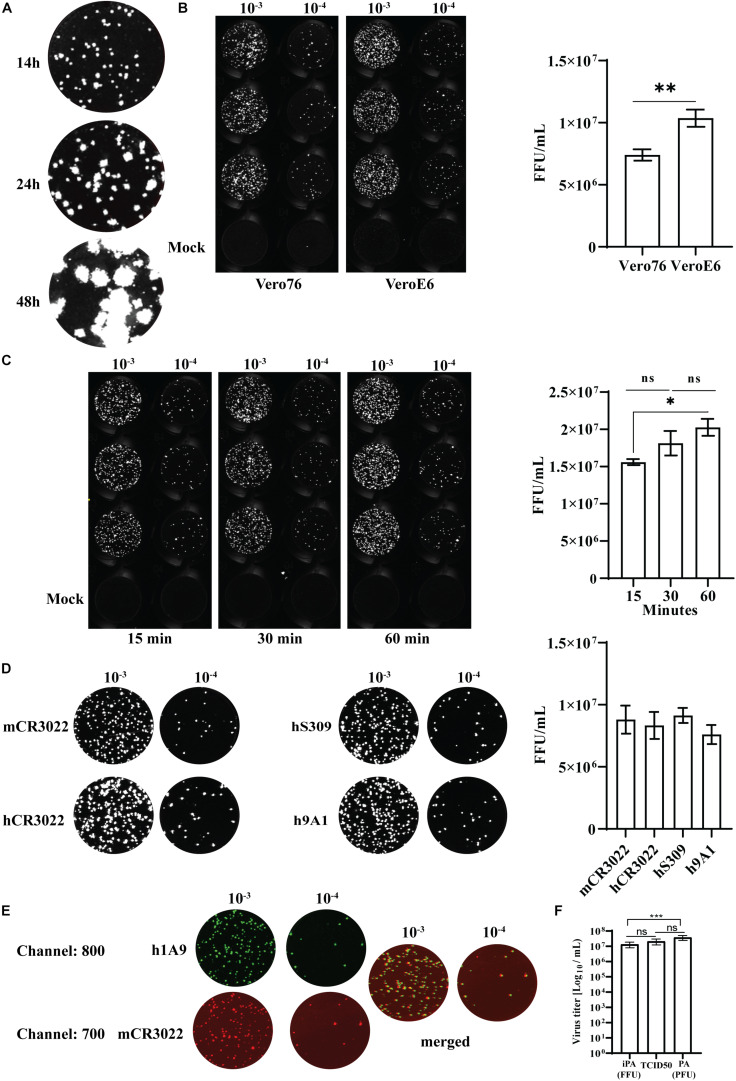
Optimization of an immuno-plaque assay for SARS-CoV-2. **(A)** A representative image of immuno-plaque sizes from infected cells fixed at different time points. **(B)** Comparison of the viral titer of the same viral stock on Vero76 and VeroE6. Left, representative images of immuno-plaque assay (iPA) performed on Vero76 and VeroE6. Right, virus titer calculated from the left. **(C)** Left, representative images of viral titers comparing different adsorption periods to infect the cells. Right, virus titer calculated from the left. **(D)** Representative images of viral titer of the same stock comparing different primary mAbs. Right, virus titer calculated from the left. **(E)** Representative images of co-staining of infected cells with two different mAbs (mCR3022 and h1A9). **(F)** Virus titer assessed by optimized iPA, TCID_50_, and standard plaque assay. The *P*-values for panels **(A)** and **(C)** (*p < 0.05, **p < 0.01) were calculated by using the Mann–Whitney *U* test. The *p*-values for panel **(F)** (***p < 0.001) were calculated by one-way analysis of variance with the Tukey multiple-comparisons test. The data presented are the mean of two or three independent experiments, where each was performed in technical duplicate or triplicate. Error bars are presented as means ± SEM.

To determine the number of foci accurately, the optimal incubation time for SARS-CoV-2-infected cells were assessed. SARS-CoV-2 (QLD02) was 10-fold serially diluted, and 25 μl of each dilution was used to infect VeroE6 for 14, 24, and 48 h post-infection (hpi) prior to fixation using a 96-well plate format. As expected, we observed that the sizes of the immuno-plaques gradually increased with time (Figure 8A), with immuno-plaque formation at 14 hpi being optimal to allow individual plaque visualization. Next, we compared whether performing iPA on Vero76 and VeroE6 can result in a comparable viral titer (Figure 8B, left). A significantly higher level of infectious virus was observed from iPA titers on VeroE6 (Figure 8B, right). Therefore, we suggest the use of VeroE6 rather than Vero76 to determine the SARS-CoV-2 viral titer by iPA to increase the assay sensitivity.

Traditionally, most methods of virus titration typically utilize a 60-min incubation period with the cell monolayer to allow sufficient time for viral adsorption (Brien et al., 2013; Agbulos et al., 2016; Mendoza et al., 2020). However, this interval is often not optimized for an individual virus and cell type and instead chosen arbitrarily as a convenient length in a laboratory setting where workers can come and go freely. As SARS-CoV-2 work has to be performed in physical lab containment level 3, where facility access can be in high demand and workers cannot readily leave and return, workflow optimization toward minimizing the assay interval time is highly advantageous. Hence, as viral adsorption to cells occurs rapidly within the first hour, we tested two periods of viral incubation – 15 and 30 min – compared to the standard duration of adsorption of 60 min (Crill and Roehrig, 2001; Roehrig et al., 2008). We found that while 15 min of incubation significantly reduced the assay sensitivity compared to the standard 60-min incubation period, 30 min of incubation period was not significantly different (Figure 8C). Thus, 30 min provides a balance between sensitivity and assay length advantage (Figure 8C).

Detection limit could also potentially vary depending on epitope availability and affinity of the primary antibody. We, therefore, compared a range of public domain mAbs that are specific for different epitopes on the SARS-CoV-2 S-protein, including CR3022 and S309 (RBD-specific mAbs) and 9A1 (S2-specific mAb) (ter Meulen et al., 2006; Chi et al., 2020; Pinto et al., 2020). Notably, there were no significant differences in titer determination among those mAbs tested, highlighting the flexibility of the iPA using different epitope-specific antibodies (Figure 8D).

The Odyssey CLx Imaging System also allows for parallel imaging of multiple targets using two different fluorescent probes. This capacity opens up the opportunity to perform multi-virus titration in a single assay. To assess the potential ability for simultaneous multi-epitope immuno-plaque detection, infected cells were co-stained with mouse CR3022 and human 9A1 mAbs, followed by two-color fluorescents (emission 680 and 800 nm) (Figure 8E). Strikingly, no significant difference (*p* = 0.17) between the two antibodies was found, showing the potential use of iPA to simultaneously target two different viral epitopes (Supplementary Material 2).

Additionally, we have compared our optimized iPA with the traditional viral titration assays such as TCID_50_ and standard plaque assay. We observed no significant differences between iPA and TCID_50_, while there was a significantly higher viral titer obtained from the standard plaque assay compared to iPA (Figure 8F). This difference is most likely due to the surface area/volume ratio of six well vs. microtiter well format (Supplementary Material 3), which allows for more viruses to be adsorbed on a per-cell basis.

### Growth Kinetics and Plaque Reduction Neutralization Assay Using a High-Throughput iPA Method

**FIGURE 9:**
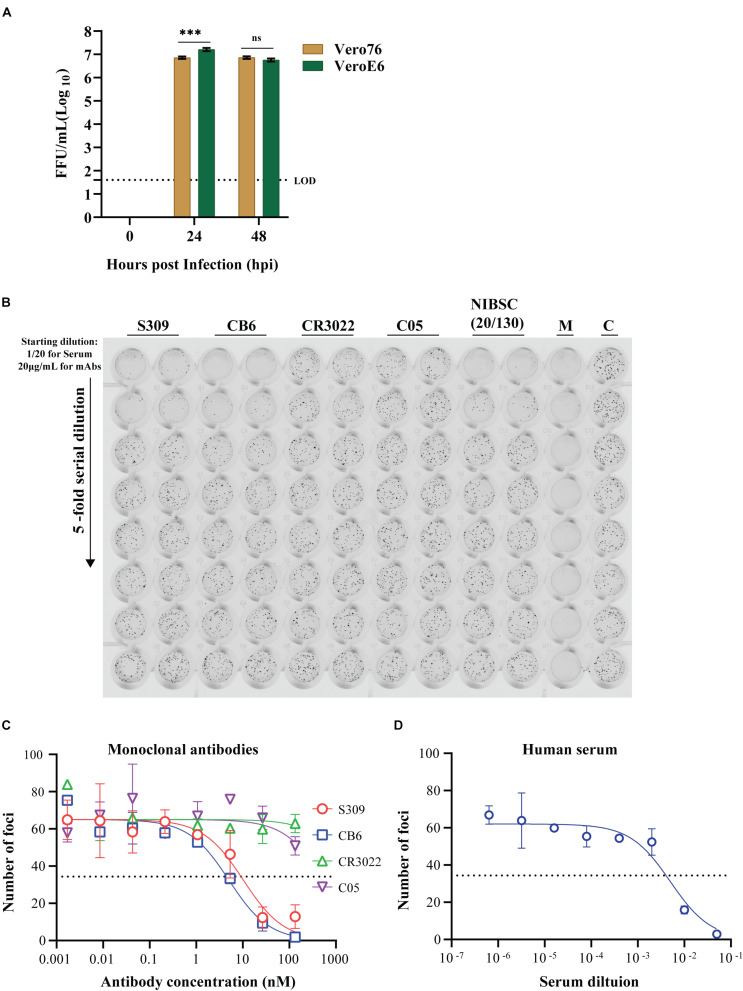
Utility of the immuno-plaque assay to determine the viral titer and neutralizing antibody levels for SARS-CoV-2 in a 96-well plate format. **(A)** Growth kinetics comparison of SARS-CoV-2 on Vero76 and VeroE6. Viral titers were determined on VeroE6. The *p*-value (***p < 0.001) for growth kinetics was determined by multiple comparison using two-way ANOVA test with Sidak’s correction. The data presented are the mean of three independent experiments, where each was performed in duplicate. Each focus counted per well of sample is then expressed as focus-forming units per milliliter (FFU/ml), and the theoretical limit of detection was determined by the detection of a single immuno-plaque in undiluted sample, which corresponds to 40 FFU/ml for a 96-well plate format. **(B)** Scanned image showing the immuno-plaques from plaque reduction neutralization test (PRNT) for mAbs and NIBSC control serum. **(C,D)** Neutralizing antibody levels for mAbs and NIBSC control serum by PRNT. The data presented is representative of three independent experiments, where each was performed in technical duplicate. The IC_50_ or ID_50_ values for each antibody and NIBSC control serum are shown in Table 1. For PRNT, to determine the IC_50_ value, the best-fit curve was tested using non-linear regression and the inhibitor vs. response (three parameters) model. The level of statistical significance was set at 95% (*p* = 0.05), and error bars are presented as means ± SEM. M and C correspond to the well containing media only or virus only, respectively.

To validate the utility of our most optimized iPA protocol (in 96-well format), growth kinetics of an Australian isolate of SARS-CoV-2 (QLD02) on VeroE6 and Vero76 cells were performed. The cells were infected at a MOI of 0.1, and infectious virus titers in the supernatant were quantified by iPA at time points 0, 24, and 48 hpi. We found that SARS-CoV-2 (QLD02) grew more efficiently on VeroE6 than Vero76, demonstrating that VeroE6 is more susceptible to SARS-CoV-2 infection (Figure 9A) or that VeroE6 is able to facilitate a higher production of SARS-CoV-2 virions compared to Vero76.

Tremendous efforts are underway in labs across the globe to develop antiviral drugs against SARS-CoV-2. To facilitate these developments, high-throughput antiviral screening methodologies are critical. With this in mind, we examined the potential for the iPA methodology to determine the level of neutralization between established anti-SARS-CoV-2 mAbs and then examined screening in a higher throughput 384-well format.

We first validated the “Protocol 2 (PRNT, in 96-well format)” by assessing the neutralizing antibody levels of four antibodies. Two of them recently published anti-SARS-CoV-2 S-protein neutralizing antibodies – S309 and CB6, a non-neutralizing S-protein antibody CR3022 and an isotype control anti-influenza HA antibody C05 (Figure 9B) (ter Meulen et al., 2006; Ekiert et al., 2012; Pinto et al., 2020; Shi et al., 2020). As expected, high levels of neutralization for S309 and CB6 antibodies were observed with inhibitory concentration (IC_50_) values of 10 and 5 nM, respectively, in comparison to CR3022 and the control C05 antibody, which display no neutralization (Figure 9C and Table 1). Additionally, a SARS-CoV-2 reference sera sample from the National Institute for Biological Standards and Control (NIBSC 20/130) was included in the assay (World Health Organization WHO, 2020d). The PRNT results using iPA showed a neutralizing level with 50% inhibitory dilution (ID_50_) of 0.005 (1/200) (Figure 9D), thereby supporting the use of iPA to determine neutralizing antibody levels.

**TABLE 1:** IC_50_ values for mAbs and human serum (National Institute for Biological Standards and Controls, NIBSC).

Sample	IC_50_ (nM)	±SEM
S309	10.2807	2.98219
CB6	5.01391	1.448
CR3022	2,444.98	4,580.11
C05	565.494	330.034
NIBSC^*a*^	0.005027 (1/200)	0.001

*^a^NIBSC is expressed as 50% inhibitory dilution.*

**FIGURE 10:**
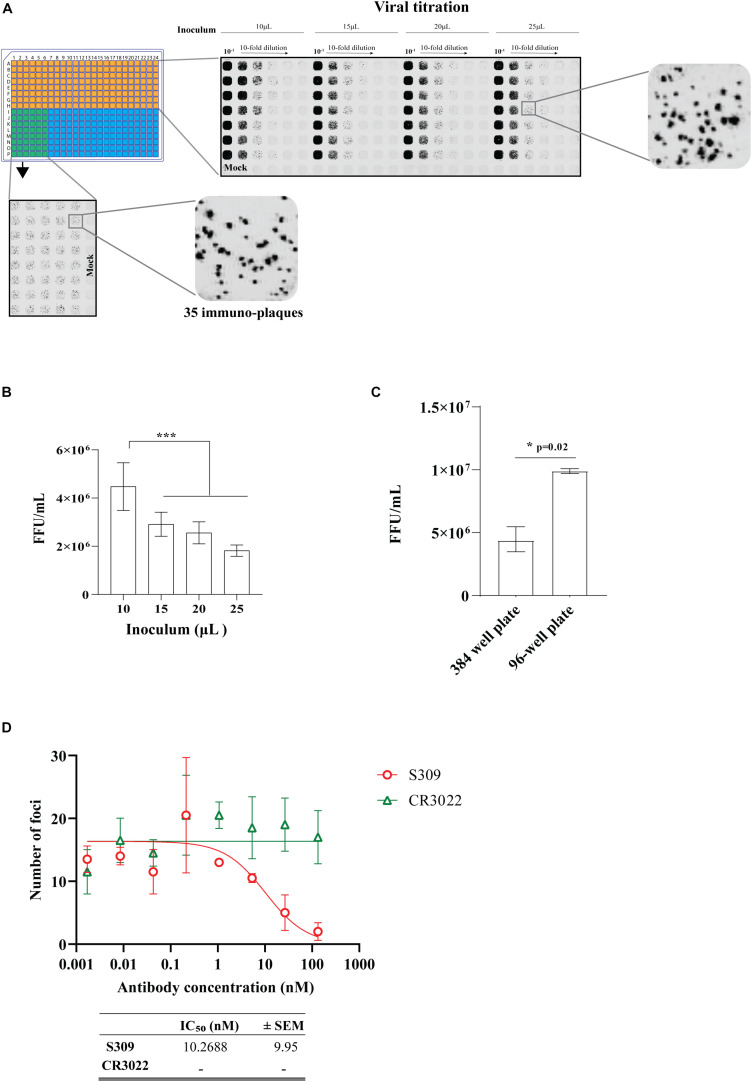
Optimization of an immuno-plaque assay for SARS-CoV-2 in 384-well plate format. **(A)** Immuno-plaque assay (iPA) showing the distinct immuno-plaque sizes from different amounts of inoculum for viral titration (top right) and for the plaque reduction neutralization test (PRNT) (bottom left). **(B)** Viral titer comparison from iPA using different amount of inoculum. The *p*-values for panel **(B)** (***p < 0.001) were calculated by one-way analysis of variance with the Tukey multiple-comparisons test. The data presented are the mean of two or three independent experiments, where each has six technical replicates. **(C)** A side-by-side comparison of viral titer assay sensitivity between 96- and 384-well plates. The *p*-value (*p < 0.05) was calculated by using the Mann–Whitney *U* test. **(D)** Curve of neutralizing antibody levels by PRNT using mAbs. The IC_50_ values for each antibody are shown in the bottom. For PRNT, to determine the IC_50_ value, the best-fit curve was tested using non-linear regression and the inhibitor vs. response (three parameters) model. The level of statistical significance was set at 95% (*p* = 0.05). Error bars are presented as means ± SEM.

Moving to the 384-well plate format, we first determined the volume of virus inoculum required to produce distinct immuno-plaques that would result in a comparable viral titer to the 96-well plate iPA format. We observed distinct and quantifiable immuno-plaques in all the volumes of virus inoculum used, including just 10 μl of virus inoculum (Figure 10A). Relevant for assay sensitivity, the virus inoculum of 10 μl resulted in a significantly higher viral titer (Figure 10B). We also determined that the optimal number of immuno-plaques for automated counting using the Virodot software was 35 per well in the 384-well format. To obtain this, 10 μl of a solution containing 6 × 10^3^ FFU/ml was mixed with another 10 μl of medium (containing 5% FCS and P/S) and incubated at 37°C for 1 h to mimic a PRNT. Then, pre-seeded VeroE6 cells were infected with 15 μl of the complex for 30 min, and subsequently an overlay was added (Figure 10A, left bottom). Next, we determined the assay sensitivity between 96- and 384-well plates by performing a side-by-side comparison of viral titers. We found that the 96-well format is a more sensitive assay than the 384-well plate (Figure 10C), which likely reflects the higher surface area/volume ratio of the 96-well vs. the 384-well format (Supplementary Material 3).

Finally, we determined the neutralization level of the S309 and CR3022 antibodies on the 384-well iPA format. We observed that the level of neutralization by the S309 antibody in the 384-well iPA format was not discernable to the level of neutralization obtained from the 96-well iPA format (Figure 10C). These results are in contrast to the assay sensitivity differences observed for viral titration between the 96- and 384-well iPA formats described above and likely reflect the quantity of virus used in each assay format, which was 150 and 90 FFU/well for the 96- and 384-well, respectively (Supplementary Material 4). These values were predetermined to produce a reproducible counting number of immuno-plaques and are sufficiently similar that they produce indistinguishable IC50 values in PRNT assays, as demonstrated for S309.

Our findings altogether indicated the flexibility of iPA to determine the virus titer and PRNT for SARS-CoV-2 from *in vitro* and *in vivo* samples. In addition, we demonstrated that iPA could be utilized and further optimized for ultra-high-throughput virus titration where samples are limited in volume and quantity, such as human sera.

## Discussion

The emergence and ongoing SARS-CoV-2 pandemic has brought together researchers from different disciplines in an effort to rapidly combat the spread of COVID-19. Accordingly, the development of a fast, reproducible, and comparable method for viral quantification, screening of antiviral agents as well as testing vaccine efficacy is of high value. Several methods for infectious SARS-CoV-2 virus detection, including standard plaque assay and TCID_50_, have been described and validated (Svensson et al., 1999; Brien et al., 2013; Smither et al., 2013; Agbulos et al., 2016; Karakus et al., 2018; Smith et al., 2019; Amanat et al., 2020; Mendoza et al., 2020). However, there are several practical limitations to these approaches, such as the associated biosafety measures, arduous experimental procedures, and experimental duration. Here we describe an alternative assay, a focus-forming assay termed iPA, for SARS-CoV-2. Most importantly, our optimized iPA provides a comparable and reliable assay for SARS-CoV-2 quantification. Furthermore, the iPA method offers advantages over traditional approaches, including a streamlined approach that is significantly faster than comparable assays, and is amenable to high-throughput capacity. An additional advantage of iPA is that it can provide both high specificity and more flexibility for virus detection.

We have previously employed the iPA methodology to determine the titers and levels of neutralizing antibodies for other pathogenic viruses such as dengue, Zika, and respiratory syncytial virus from *in vitro* and *in vivo* experimental samples (Faddy et al., 2016; Watterson et al., 2016; Jaberolansar et al., 2017; Li et al., 2018; Modhiran et al., 2019; Setoh et al., 2019; Slonchak et al., 2020). To adapt iPA to determine the titer of SARS-CoV-2 in samples, we have optimized the parameters, including the duration of virus adsorption and infection. Similar to the standard plaque assay and TCID_50_, SARS-CoV-2 is allowed to adsorb onto a cell monolayer followed by an additional overlay of semisolid media. During incubation, the infected cells release virus, which spreads iteratively to neighboring cells, forming a circular zone that can be visualized by virus-specific antibodies. Each focus is considered to be the product of a single virus particle. As observed in Figures 8A,C, distinct and quantifiable immuno-plaques were observed using 30 min of adsorption period, and 14 h of infection was comparable to the standard duration of adsorption of 1 h; hence, the iPA assay requires 24 h from adsorption to completion. Therefore, in comparison to TCID_50_ (3 days) and the standard plaque assay (3–4 days), this allows a significant reduction in the time taken to complete the virus titration. Additionally, iPA yielded similar virus titers compared to the TCID_50_ method (Figure 8F), demonstrating that sensitivity is not compromised using the shorter assay format. However, we found a significantly higher viral titer using the standard plaque assay compared to iPA. This is most likely due to the higher surface area/volume ratio of the six-well vs. 96-well format, which increases relative viral contact with the cells in the larger-well format and leads to increased assay sensitivity (Supplementary Material 3), with the drawback of larger assay footprint and longer assay time.

We demonstrated the flexibility of iPA detection by using several S protein-specific antibodies and found reliable detection across domain-specific antibodies (Figure 8C) (ter Meulen et al., 2006; Chi et al., 2020; Pinto et al., 2020). The variable domain sequences of these antibodies are in the public domain, and they can be readily produced recombinantly in labs equipped with appropriate facilities and access to commercial gene synthesis. Alternatively, the results suggest that many of the major epitopes are well preserved using our fixation method, and the format would likely work well using monoclonal and polyclonal preparations made in-house by individual laboratories. Furthermore, dual-probing with two distinct epitope-specific antibodies does not impact on viral titer determination and can be used for co-staining application (Figure 8C). This provides a benefit of iPA over the standard plaque assay and TCID_50_, with the potential to investigate co-infection by different viruses simultaneously. Additionally, a combination of immunostaining and plaque assay technique could facilitate researchers to precisely identify and quantify viral infections from clinical samples.

Currently, VeroE6 remains the cell line of choice in many virus titration assays for SARS-CoV-2, but no experiments were conducted to assess and determine if VeroE6 is, in fact, the best cell line for SARS-CoV-2 titration. Therefore, we attempted to identify and understand the factors behind the extensive usage of VeroE6 for SARS-CoV-2 titration by performing multi-cycle growth kinetics to compare the permissiveness of VeroE6 and another variant of Vero cell line, Vero76, on supporting SARS-CoV-2 replication and virion production. Both VeroE6 and Vero76 clones are well-established cell lines for the manufacture of human vaccines and are typically a first-choice cell line to use for *in vitro* assays of several highly pathogenic viruses such as Ebola virus, influenza virus, Middle East respiratory syndrome coronavirus, and SARS-CoV-2 (Barrett et al., 2009; Montomoli et al., 2012; Zaki et al., 2012; Castilletti et al., 2015; Harcourt et al., 2020). From our growth kinetics, we observed that SARS-CoV-2 replicated more efficiently on VeroE6 compared to Vero76 over a 48-h period. In agreement with our observation, a recent study reported slightly higher viral titers in VeroE6 cells when compared to Vero CCL81 cells (Harcourt et al., 2020). Taking into consideration that (i) several studies have reported that SARS-CoV-2 utilizes the ACE2 and transmembrane serine protease 2 (TMPRSS2) expressed on the cell surface to establish infection (Li et al., 2003; Simmons et al., 2004; Hoffmann et al., 2020; Walls et al., 2020), (ii) ACE2 is expressed highly in VeroE6 cells (Ren et al., 2006), and (iii) the critical role of TMPRSS2 for viral infectivity (Matsuyama et al., 2020), we hypothesized that SARS-CoV-2 can replicate more efficiently on VeroE6 due to the differential expression of TMPRSS2 between VeroE6 and Vero76 clones (Ren et al., 2006; Matsuyama et al., 2020).

Determining antibody neutralizing titer is a key criterion for the successful development of effective antivirals and vaccines. PRNT is commonly used to assess the level of neutralizing antibodies. A classic approach is to use an agarose overlay in six-well plate formats, which necessitates the consumption of a significant amount of resources, samples, and longer incubation times, resulting in a more expensive, laborious and time-intensive assay which is difficult to perform at higher throughput levels in a biosafety level 3 laboratory. Alternatively, a high-throughput assay such as the TCID_50_ assay has been extensively described for several viruses, including SARS-CoV-2 (Svensson et al., 1999; Smither et al., 2013; Karakus et al., 2018; Smith et al., 2019; Amanat et al., 2020). Here we have optimized a high throughput iPA that is faster than TCID_50_, with greater accuracy due to plaque readouts versus absorbance readouts. We have demonstrated how iPA can be used to analyze the neutralization levels for SARS-CoV-2 in both 96- and 386-well formats. Two potent antibodies (S309 and CB6) that target the SARS-CoV-2 spike protein and efficiently neutralize SARS-CoV-2 were included in this study (Pinto et al., 2020; Shi et al., 2020). We found that 50% inhibitory concentration (IC_50_) is at 10 nM (1.3 ug/ml) and 5 nM (0.83 ug/ml), respectively. Our neutralization assay for S309 showed a slightly lower potency compared to a previous report (Pinto et al., 2020). This is likely attributed to the input (amount/strain) viruses incubated with antibody and the different approaches. Consistent with previous studies, no neutralization was observed from CR3022 mAb (ter Meulen et al., 2006). Additionally, we reported NISBC control sera in this assay demonstrating 50% inhibitory dilution (ID_50_) of 0.005 (1/200); we hope this could set a reference to those interested in performing iPAs.

Many researchers around the world are limited by the volumes they can secure of precious samples such as serum or antibodies for viral titration or PRNT. To overcome this problem, we further optimized the iPA on 384-well plates by using only 10 ul of the sample for virus titration and 15 ul for PRNT. As expected, viral titers on 384-well plates were lower when compared to iPA on 96-well plates (Figure 10C). However, due to the relatively similar input virus levels in the PRNT assay setup, we observed comparable neutralizing antibody levels between 96- and 384-well plates. Thus, while the 96-well iPA format offers advantages in sensitivity and may be preferable to assess low-titer sera samples, validation that the 384-well format delivers similar PRNT IC50 values provides a robust high-throughput format for researchers to rapidly screen antiviral compounds against live virus.

Although the iPA is an efficient high-throughput screening method, there are several limitations. Specifically, specific antibodies and equipment (Odyssey scanner) are required for visualization. Additionally, distinct and clear foci must be obtained. Despite these limitations, the iPA provides an alternative and complementary method for quantification and antiviral screening against SARS-CoV-2.

## Conclusion

The most critical factor for successful, reliable, and efficient virus titration and neutralization assay lies in the optimization of the protocol. Here, we described an optimized iPA as a reliable method to enable the high-throughput study of SARS-CoV-2 infection and to enhance the rate of discovery and improve the feasibility of large-scale screens. We demonstrated that the iPA assay is an efficient means of determining viral titer and can be utilized as a platform for high-throughput screening of SARS-CoV-2 infectivity and neutralization, which has applications for therapeutic and vaccine development.

## Data Availability Statement

The original contributions presented in the study are included in the article/Supplementary Material, further inquiries can be directed to the corresponding author/s.

## Author Contributions

AAA, NM, KJC, PRY, AAK, and DW have contributed to the experimental design. AAA, NM, YXS, NYGP, JDJS, BL, CLDM, MEF, and STMC have performed experiments. AAA, NM, YXS, NYGP, CLDM, MEF, AAK, and DW wrote the initial drafts of the manuscript. AAA, NM, and DW have performed the data analysis. All the authors contributed to editing of the manuscript.

## Conflict of Interest

The authors declare that the research was conducted in the absence of any commercial or financial relationships that could be construed as a potential conflict of interest.

## Supplementary Material

Supplementary Material 1 Summaries for protocols 1 and 2.

Supplementary Material 2 Virus titer assessed by optimized immuno-plaque assay. The infected cells were co-stained with mouse CR3022 and human 9A1 mAbs using two alternate fluorophore conjugates and secondary antibody probes (emission, 680 and 800 nm). Individual immuno-plaque values for each condition were counted, and no significant difference between the two antibodies was found using the Mann–Whitney *U* test. The data presented are the mean of two independent experiments, where each was performed in duplicate. The level of statistical significance was set at 95% (*p* = 0.05), and error bars are presented as means ± SEM.

Supplementary Material 3 Calculation of surface area/volume ratio for different plate formats.

Supplementary Material 4 Calculation of viral amount used for different plate formats.

## References

[B1] Agbulos D. S. Barelli L. Giordano B. V. Hunter F. F. (2016). Zika virus: quantification, propagation, detection, and storage. *Curr. Protoc. Microbiol.* 43 15D.4 1–15D.16. 10.1002/cpmc.19 27858969

[B2] Amanat F. White K. M. Miorin L. Strohmeier S. McMahon M. Meade P. (2020). An in vitro microneutralization assay for SARS-CoV-2 serology and drug screening. *Curr. Protoc. Microbiol.* 58:e108. 10.1002/cpmc.108 PMC7361222 32585083

[B3] Barrett P. N. Mundt W. Kistner O. Howard M. K. (2009). Vero cell platform in vaccine production: moving towards cell culture-based viral vaccines. *Expert Rev. Vaccines* 8 607–618. 10.1586/erv.09.19 19397417

[B4] Brien J. D. Lazear H. M. Diamond M. S. (2013). Propagation, quantification, detection, and storage of west nile virus. *Curr. Protoc. Microbiol.* 31 15D.3.1–15D.3.18. 10.1002/9780471729259.mc15d03s31 24510289

[B5] Castilletti C. Carletti F. Gruber C. E. Bordi L. Lalle E. Quartu S. (2015). Molecular characterization of the first ebola virus isolated in italy, from a health care worker repatriated from sierra leone. *Genome Announc.* 3 e639–e615. 10.1128/genomeA.00639-15 PMC4472897 26089420

[B6] Chi X. Yan R. Zhang J. Zhang G. Zhang Y. Hao M. (2020). A neutralizing human antibody binds to the N-terminal domain of the Spike protein of SARS-CoV-2. *Science* 369 650–655. 32571838 10.1126/science.abc6952 PMC7319273

[B7] Crill W. D. Roehrig J. T. (2001). Monoclonal antibodies that bind to domain III of dengue virus E glycoprotein are the most efficient blockers of virus adsorption to Vero cells. *J. Virol.* 75 7769–7773. 10.1128/jvi.75.16.7769-7773.2001 11462053 PMC115016

[B8] Ekiert D. C. Kashyap A. K. Steel J. Rubrum A. Bhabha G. Khayat R. (2012). Cross-neutralization of influenza a viruses mediated by a single antibody loop. *Nature* 489 526–532. 10.1038/nature11414 22982990 PMC3538848

[B9] Faddy H. M. Fryk J. J. Watterson D. Young P. R. Modhiran N. Muller D. A. (2016). Riboflavin and ultraviolet light: impact on dengue virus infectivity. *Vox Sang* 111 235–241. 10.1111/vox.12414 27281512

[B10] Harcourt J. Tamin A. Lu X. Kamili S. Sakthivel S. K. Murray J. (2020). Severe acute respiratory syndrome Coronavirus 2 from patient with Coronavirus disease, United States. *Emerg. Infect. Dis.* 26 1266–1273. 32160149 10.3201/eid2606.200516 PMC7258473

[B11] Hoffmann M. Kleine-Weber H. Schroeder S. Kruger N. Herrler T. Erichsen S. (2020). SARS-CoV-2 cell entry depends on ACE2 and TMPRSS2 and Is blocked by a clinically proven protease inhibitor. *Cell* 181:e8. 10.1016/j.cell.2020.02.052 PMC7102627 32142651

[B12] Jaberolansar N. Chappell K. J. Watterson D. Bermingham I. M. Toth I. Young P. R. (2017). Induction of high titerd, non-neutralising antibodies by self-adjuvanting peptide epitopes derived from the respiratory syncytial virus fusion protein. *Sci. Rep.* 7:11130. 10.1038/s41598-017-10415-w PMC5593926 28894111

[B13] Jones M. L. Seldon T. Smede M. Linville A. Chin D. Y. Barnard R. (2010). A method for rapid, ligation-independent reformatting of recombinant monoclonal antibodies. *J. Immunol. Methods* 354 85–90. 10.1016/j.jim.2010.02.001 20153332

[B14] Karakus U. Crameri M. Lanz C. Yanguez E. (2018). Propagation and titration of influenza viruses. *Methods Mol. Biol.* 1836 59–88. 10.1007/978-1-4939-8678-1_4 30151569

[B15] Katzelnick L. C. Coello Escoto A. McElvany B. D. Chavez C. Salje H. Luo W. (2018). Viridot: an automated virus plaque (immunofocus) counter for the measurement of serological neutralizing responses with application to dengue virus. *PLoS Negl. Trop. Dis.* 12:e0006862. 10.1371/journal.pntd.0006862 30356267 PMC6226209

[B16] Lan J. Ge J. Yu J. Shan S. Zhou H. Fan S. (2020). Structure of the SARS-CoV-2 spike receptor-binding domain bound to the ACE2 receptor. *Nature* 581 215–220. 32225176 10.1038/s41586-020-2180-5

[B17] Li J. Watterson D. Chang C. W. Che X. Y. Li X. Q. Ericsson D. J. (2018). Structural and functional characterization of a cross-reactive dengue virus neutralizing antibody that recognizes a cryptic epitope. *Structure* 26:e4. 10.1016/j.str.2017.11.017 29249606

[B18] Li W. Moore M. J. Vasilieva N. Sui J. Wong S. K. Berne M. A. (2003). Angiotensin-converting enzyme 2 is a functional receptor for the SARS coronavirus. *Nature* 426 450–454. 14647384 10.1038/nature02145 PMC7095016

[B19] Madu I. G. Roth S. L. Belouzard S. Whittaker G. R. (2009). Characterization of a highly conserved domain within the severe acute respiratory syndrome coronavirus spike protein S2 domain with characteristics of a viral fusion peptide. *J. Virol.* 83 7411–7421. 10.1128/jvi.00079-09 19439480 PMC2708636

[B20] Matsuyama S. Nao N. Shirato K. Kawase M. Saito S. Takayama I. (2020). Enhanced isolation of SARS-CoV-2 by TMPRSS2-expressing cells. *Proc. Natl. Acad. Sci. U S A.* 117 7001–7003. 10.1073/pnas.2002589117 32165541 PMC7132130

[B21] Mendoza E. J. Manguiat K. Wood H. Drebot M. (2020). Two detailed plaque assay protocols for the quantification of infectious SARS-CoV-2. *Curr. Protoc. Microbiol.* 57:ecmc105. 10.1002/cpmc.105 PMC7300432 32475066

[B22] Modhiran N. Gandhi N. S. Wimmer N. Cheung S. Stacey K. Young P. R. (2019). Dual targeting of dengue virus virions and NS1 protein with the heparan sulfate mimic PG545. *Antiviral Res.* 168 121–127. 10.1016/j.antiviral.2019.05.004 31085206

[B23] Montomoli E. Khadang B. Piccirella S. Trombetta C. Mennitto E. Manini I. (2012). Cell culture-derived influenza vaccines from Vero cells: a new horizon for vaccine production. *Expert Rev. Vaccines* 11 587–594. 10.1586/erv.12.24 22827244

[B24] Pinto D. Park Y. J. Beltramello M. Walls A. C. Tortorici M. A. Bianchi S. (2020). Cross-neutralization of SARS-CoV-2 by a human monoclonal SARS-CoV antibody. *Nature* 583 290–295. 32422645 10.1038/s41586-020-2349-y

[B25] ProMED (2019). *Undiagnosed Pneumonia - China (HU). ProMED International Society for Infectious Diseases.* Available online at: https://promedmail.org/promed-post/?id=6864153%20#COVID19 (accessed October 10, 2020).

[B26] Reed L. Muench H. (1938). A simple method of estimating fifty per cent endpoints. *Am. J. Epidemiol. Vol.* 27 493–497. 10.1093/oxfordjournals.aje.a118408

[B27] Ren X. Glende J. Al-Falah M. de Vries V. Schwegmann-Wessels C. Qu X. (2006). Analysis of ACE2 in polarized epithelial cells: surface expression and function as receptor for severe acute respiratory syndrome-associated coronavirus. *J. Gen. Virol.* 87 1691–1695. 10.1099/vir.0.81749-0 16690935

[B28] Roehrig J. T. Hombach J. Barrett A. D. (2008). Guidelines for plaque-reduction neutralization testing of human antibodies to dengue viruses. *Viral Immunol.* 21 123–132. 10.1089/vim.2008.0007 18476771

[B29] Setoh Y. X. Amarilla A. A. Peng N. Y. G. Griffiths R. E. Carrera J. Freney M. E. (2019). Determinants of Zika virus host tropism uncovered by deep mutational scanning. *Nat. Microbiol.* 4 876–887. 10.1038/s41564-019-0399-4 30886357 PMC6667831

[B30] Shi R. Shan C. Duan X. Chen Z. Liu P. Song J. (2020). A human neutralizing antibody targets the receptor-binding site of SARS-CoV-2. *Nature* 584 120–124. 32454512 10.1038/s41586-020-2381-y

[B31] Simmons G. Reeves J. D. Rennekamp A. J. Amberg S. M. Piefer A. J. Bates P. (2004). Characterization of severe acute respiratory syndrome-associated coronavirus (SARS-CoV) spike glycoprotein-mediated viral entry. *Proc. Natl. Acad. Sci. U S A.* 101 4240–4245. 10.1073/pnas.0306446101 15010527 PMC384725

[B32] Slonchak A. Hugo L. E. Freney M. E. Hall-Mendelin S. Amarilla A. A. Torres F. J. (2020). Zika virus noncoding RNA suppresses apoptosis and is required for virus transmission by mosquitoes. *Nat. Commun.* 11:2205. 10.1038/s41467-020-16086-y PMC7200751 32371874

[B33] Smith M. R. Schirtzinger E. E. Wilson W. C. Davis A. S. (2019). Rift valley fever virus: propagation. quantification, and storage. *Curr. Protoc. Microbiol.* 55:e92. 10.1002/cpmc.92 31763765

[B34] Smither S. J. Lear-Rooney C. Biggins J. Pettitt J. Lever M. S. Olinger G. G. Jr. (2013). Comparison of the plaque assay and 50% tissue culture infectious dose assay as methods for measuring filovirus infectivity. *J. Virol. Methods* 193 565–571. 10.1016/j.jviromet.2013.05.015 23748121

[B35] Svensson L. Hjalmarsson A. Everitt E. (1999). TCID50 determination by an immuno dot blot assay as exemplified in a study of storage conditions of infectious pancreatic necrosis virus. *J. Virol. Methods* 80 17–24. 10.1016/s0166-0934(99)00018-x 10403672

[B36] ter Meulen J. van den Brink E. N. Poon L. L. Marissen W. E. Leung C. S. Cox F. (2006). Human monoclonal antibody combination against SARS coronavirus: synergy and coverage of escape mutants. *PLoS Med.* 3:e237. 10.1371/journal.pmed.0030237 16796401 PMC1483912

[B37] Walls A. C. Park Y. J. Tortorici M. A. Wall A. McGuire A. T. Veesler D. (2020). Structure. function, and antigenicity of the SARS-CoV-2 spike glycoprotein. *Cell* 181 281–292.e6. 32155444 10.1016/j.cell.2020.02.058 PMC7102599

[B38] Watterson D. Robinson J. Chappell K. J. Butler M. S. Edwards D. J. Fry S. R. (2016). A generic screening platform for inhibitors of virus induced cell fusion using cellular electrical impedance. *Sci. Rep.* 6:22791. 10.1038/srep22791 PMC4792136 26976324

[B39] World Health Organization WHO (2020a). *Coronavirus Disease (COVID-19) Dashboard.* Geneva: World Health Organization.

[B40] World Health Organization WHO (2020b). *Naming the Coronavirus Disease (COVID-19) and the Virus that Causes it. World Health Organization (WHO) Technical guidance.* Geneva: World Health Organization.

[B41] World Health Organization WHO (2020c). *Press Briefing on WHO Mission to China and Novel Coronavirus Outbreak. World Health Organization (WHO), General Director Speeches.* Geneva: World Health Organization.

[B42] World Health Organization WHO (2020d). *Research Reagent for Anti-SARS-CoV-2 Ab NIBSC code 20/130.* Geneva: World Health Organization.

[B43] Wrapp D. Wang N. Corbett K. S. Goldsmith J. A. Hsieh C. L. Abiona O. (2020). Cryo-EM structure of the 2019-nCoV spike in the prefusion conformation. *Science* 367 1260–1263. 32075877 10.1126/science.abb2507 PMC7164637

[B44] Wu F. Zhao S. Yu B. Chen Y. M. Wang W. Song Z. G. (2020). A new coronavirus associated with human respiratory disease in China. *Nature* 579 265–269. 32015508 10.1038/s41586-020-2008-3 PMC7094943

[B45] Wu Z. McGoogan J. M. (2020). Characteristics of and important lessons from the Coronavirus Disease 2019 (COVID-19) outbreak in china: summary of a report of 72314 cases from the chinese center for disease control and prevention. *JAMA* 323 1239–1242. 10.1001/jama.2020.2648 32091533

[B46] Zaki A. M. van Boheemen S. Bestebroer T. M. Osterhaus A. D. Fouchier R. A. (2012). Isolation of a novel coronavirus from a man with pneumonia in Saudi Arabia. *N. Engl. J. Med.* 367 1814–1820. 10.1056/nejmoa1211721 23075143

[B47] Zhou P. Yang X. L. Wang X. G. Hu B. Zhang L. Zhang W. (2019). A pneumonia outbreak associated with a new coronavirus of probable bat origin. *Nature* 579 270–273. 10.1038/s41586-020-2012-7 32015507 PMC7095418

