# Supplementary material for: An Optimized High-Throughput Immuno-Plaque Assay for SARS-CoV-2
Source: Front Microbiol. 2021 Feb 12;12:625136. doi: 10.3389/fmicb.2021.625136 (PMC7906992; doi:10.3389/fmicb.2021.625136)
Supplement: Supplementary Material 1 — Summaries for protocols 1 and 2. [file Data_Sheet_1.docx]

**Supplementary Material 1. Summaries for Protocol 1 and 2.**

**Protocol 1, (iPA)**

Briefly, Vero (76 or E6) at density cells of approximately 4×10^4^ or 2×10^4^ cells per well were seeded in a 96 or 384 well plates, respectively and incubated for 14 h to reach 100 % confluency. Virus-containing samples were serially diluted 10-fold in DMEM supplemented with 2 % FCS and P/S. Culture media was then removed from the cells and immediately incubated with 25 μL or 10 μL of the virus sample for 96 or 384 well plates, respectively. Following 30 min of incubation, 175 μL or 50 μL of overlay medium was added to 96 or 384 well plates, respectively. After 14 h of infection, the overlay was removed by using a multichannel pipette and the entire plate was immediately submerged in a container with cold 80 % acetone and kept for 30 min at -20 ⁰C. Subsequently, the plate was fully dried for 2 h by using the biosafety cabinet fan. Once all the fixative has evaporated, the plate was probed for viral proteins. First, the plate was blocked for 60 min at 37 °C by adding 150 μL or 50 μL of blocking solution (KPL Milk Diluent/Blocking Solution Concentrate, Sera care, USA) to 96 or 384 well plates, respectively. After which, the respective wells of the plate were probed with primary mAb for 1 h at 37 °C, using 50 μl or 20 μl/well (50 or 20 μg/well) for 96 and 384-well plates, respectively. Plate was washed five times with PBS containing 0.05 % Tween 20. Subsequently, a fluorophore conjugated secondary antibody (IRDye, LI-COR, USA) was added and incubated for 1 h at 37 °C. For secondary antibody, we used 50 or 20 μl/well (20 or 8 ng/well) for 96 and 384-well plates, respectively. The monolayer was then washed five times and fully dried while avoiding light exposure. The respective dried plates were scanned using the LI-COR Biosciences Odyssey Infrared Imaging System (Odessey CLx, Li-COR, USA). Each foci counted per well of sample is then expressed as focus forming units per milliliter, or FFU/mL and the theoretical limit of detection (LOD) was determined by the detection of a single immuno-plaque in undiluted sample, which correspond to 40 FFU/mL and 100 FFU/mL for 96 and 384-wells respectively.

**Protocol 2, Plaque Reduction Neutralisation Test (PRNT)**

A detailed **protocol 2** for PRNT is provided in the method section in the main manuscript. To validate our PRNT protocol using iPA, the level of neutralising antibodies present in human serum was assessed against the National Institute for Biological Standards and Control (NIBSC) control sera for SARS-CoV-2 [27]. Additionally, neutralisation levels of different human monoclonal antibody (mAb) against SARS-CoV-2, including S309, CB6 and CR3022 were tested. A mAb against Hemagglutination Influenza A (anti-HA antibody, C05) was used as an isotype control antibody. Briefly, serum was heat-inactivated at 56 °C for 30 minutes, and five-fold serial dilutions were performed in DMEM containing 5% FCS and antibiotic. For serum and mAbs, starting dilutions were 1/20 and 20 µg/mL, respectively. Following, each dilution of serum or mAb, samples were incubated with SARS-CoV-2 (QLD02) and incubated at 37 °C for 1 h. Subsequently, the mixture of virus and antibody/sera complex (50 µL for 96-well plate or 15 µL for 384-well plate) was added onto VeroE6 cells (4 x10^4^ or 2 x10^4^ cells per well in 96 or 384-well plates, respectively) and incubated for 30 min at 37 °C. Then, the inoculum was kept on the cells, overlay medium added, and the plates were incubated at 37 °C with 5% of CO_2_ for 14 h. The cells were fixed and probed as described above, using mouse CR3022 as the primary antibody. Immuno-plaques were counted using an automated virus plaque counter program, Viridot [28].
