# Supplementary figures and images for: An Optimized High-Throughput Immuno-Plaque Assay for SARS-CoV-2
Source: Front Microbiol. 2021 Feb 12;12:625136. doi: 10.3389/fmicb.2021.625136 (PMC7906992; doi:10.3389/fmicb.2021.625136)

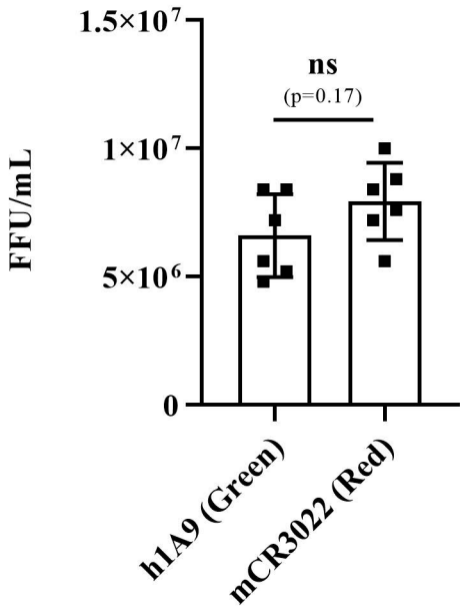

Supplement: Supplementary Material 2 — Virus titer assessed by optimized immuno-plaque assay. The infected cells were co-stained with mouse CR3022 and human 9A1 mAbs using two alternate fluorophore conjugates and secondary antibody probes (emission, 680 and 800 nm). Individual immuno-plaque values for each condition were counted, and no significant difference between the two antibodies was found using the Mann–Whitney U test. The data presented are the mean of two independent experiments, where each was performed in duplicate. The level of statistical significance was set at 95% (p = 0.05), and error bars are presented as means ± SEM. [file Data_Sheet_2.pdf]
