# Supplementary material for: An Optimized High-Throughput Immuno-Plaque Assay for SARS-CoV-2
Source: Front Microbiol. 2021 Feb 12;12:625136. doi: 10.3389/fmicb.2021.625136 (PMC7906992; doi:10.3389/fmicb.2021.625136)
Supplement: Supplementary Material 3 — Calculation of surface area/volume ratio for different plate formats. [file Data_Sheet_3.docx]

| **Format of Plate wells** | **6** | **96** | **384** |
| --- | --- | --- | --- |
| **well area (cm^2^)** | 9.6 | 0.32 | 0.056 |
| **infection volume (ml)** | 0.2 | 0.025 | 0.010 |
| **Surface area/volume Ratio** | 48 | 12.8 | 3.733 |

**Supplementary Material 3.** Calculation of surface area/volume ratio for different plate format.
