# Supplementary material for: An Optimized High-Throughput Immuno-Plaque Assay for SARS-CoV-2
Source: Front Microbiol. 2021 Feb 12;12:625136. doi: 10.3389/fmicb.2021.625136 (PMC7906992; doi:10.3389/fmicb.2021.625136)
Supplement: Supplementary Material 4 — Calculation of viral amount used for different plate formats. [file Data_Sheet_4.docx]

**Supplementary Material 4.** Calculation of viral amount used for different plate format.

| **PRNT Plate Format** | **96 well** | **384 well** |
| --- | --- | --- |
| **Input virus titre (FFU/ml)** | 3.00E+03 | 6.00E+03 |
| **Inoculum volume (ml)** | 0.05 | 0.015 |
| **FFU/well** | 150 | 90 |
